# Taiwan Bat Lyssavirus: In Vitro and In Vivo Assessment of the Ability of Rabies Vaccine-Derived Antibodies to Neutralise a Novel Lyssavirus

**DOI:** 10.3390/v14122750

**Published:** 2022-12-09

**Authors:** Rebecca Shipley, Edward Wright, Samuel P. Smith, David Selden, Anthony R. Fooks, Ashley C. Banyard

**Affiliations:** 1 Wildlife Zoonoses and Vector-Borne Diseases Research Group, Animal and Plant Health Agency (APHA), Weybridge, London KT15 3NB, UK; 2 Viral Pseudotype Unit, School of Life Sciences, University of Sussex, Falmer, Brighton BN1 9QG, UK; 3 Institute for Infection and Immunity, St. George’s Hospital Medical School, University of London, London SW17 0RE, UK

**Keywords:** rabies, lyssavirus, Taiwan bat lyssavirus, TWBLV, bats, vaccine protection, neutralisation, emerging, novel, zoonoses

## Abstract

Rabies is a neglected tropical disease. The prototype virus, the rabies virus, still causes tens of thousands of human fatalities annually. Rabies is one member of the genus Lyssavirus. The burden of other lyssaviruses is unclear. The continued emergence of novel lyssaviruses means that assessment of vaccine efficacy against these viruses is critical, as standard rabies vaccines are not efficacious against all lyssaviruses. Taiwan bat lyssavirus (TWBLV) was first reported in 2018 following isolation from Japanese house bats. Since the initial detection and genetic characterisation, no attempts have been made to antigenically define this virus. Due to the inaccessibility of the wildtype isolate, the successful generation of a live recombinant virus, cSN-TWBLV, is described, where the full-length genome clone of the RABV vaccine strain, SAD-B19, was constructed with the glycoprotein of TWBLV. In vitro and in vivo characterization of cSN-TWBLV was undertaken and demonstrated evidence for cross-neutralisation of cSN-TWBLV with phylogroup I -specific sera and rabies virus standard sera. For neutralisation equivalent to 0.5 IU/mL of WHO and World Organisation of Animal Health (WOAH) sera against CVS, 0.5 IU/mL of WOAH sera and 2.5 IU/mL of WHO sera were required to neutralise cSN-TWBLV. In addition, specific sera for ARAV and EBLV-1 exhibited the highest neutralising antibody titres against cSN-TWBLV, compared to other phylogroup I-specific sera.

## 1. Introduction

Rabies is caused by rabies virus (RABV) and related viral species within an emerging genus of viruses termed lyssaviruses [1]. The continued emergence of lyssaviruses is of significance to human populations, as the lyssaviruses are invariably associated with fatality following the onset of symptoms [2]. Within the genus, RABV is the most important lyssavirus from global animal and human health perspectives and is purported to cause tens of thousands of deaths annually, with the majority of these deaths occurring in Africa and Asia, where rabies is endemic in dog populations. However, all lyssaviruses are capable of causing rabies. As such, interest in lyssaviruses, especially those discovered following emergence genetically or antigenically divergent to RABV, is reinvigorated with each newly discovered viral species [3]. The lyssavirus genus, within the family Rhabdoviridae, comprises of an increasing number of characterised and classified viruses. In the past two decades, nine of the 17 recognized lyssaviruses have been detected, as well as two novel tentative virus species that remain to be formally classified within the genus [4,5,6]. The burden of the non-RABV lyssaviruses is undefined; only 14 human lyssavirus-related deaths have been reported previously [7,8]. This statistic is likely not representative of the true burden of infection as the diagnostic capabilities in endemic areas, often limited to clinical diagnosis and antigen detection, are unable to distinguish between lyssavirus species [7,9].

In addition to genetic classification into species, lyssaviruses have been assigned to three distinct phylogroups based on immunological, pathological, and genetic characteristics [10,11]. Rabies vaccines marketed for humans confer protective immunity against phylogroup I lyssaviruses, which include: Australian bat lyssavirus (ABLV), Aravan lyssavirus (ARAV), Bokeloh bat lyssavirus (BBLV), Duvenhage lyssavirus (DUVV), European bat-1 lyssavirus (EBLV-1), European bat-2 lyssavirus (EBLV-2), Gannoruwa bat lyssavirus (GBLV), Irkut Lyssavirus (IRKV), Khujand Lyssavirus (KHUV), and RABV [12,13,14]. Vaccination and challenge studies in animal models utilizing existing rabies vaccines have determined that the antibody titre induced in the recipient of the vaccine directly correlates to the immunity against phylogroup I lyssaviruses. Whilst an antibody titre of 0.5 IU/mL has been previously associated with an increased likelihood of cross-protection against classical RABV, evidence suggests that higher neutralizing antibody titres are required against non-RABV phylogroup I viruses [12,15,16,17]. In contrast, an assessment of vaccine efficacy demonstrated that existing vaccines are unable to confer protection against the lyssavirus species in phylogroup II (Lagos bat virus, Mokola virus, and Shimoni bat virus) and phylogroup III (Ikoma lyssavirus, Lleida bat lyssavirus, and West Caucasian bat virus) [18,19,20].

In 2016 and 2017, a novel lyssavirus was detected in two Japanese pipistrelles (*Pipistrellus abramus*) in Tainan City and Yunlin County. These were named Taiwan Bat Lyssavirus (TWBLV) after the virus was isolated and genomic sequences for each of the viral proteins showed that it was a distinct lyssavirus species [21]. Genetically, TWBLV is closely related to EBLV1 (75%) and IRKV (74%) when comparing the concatenated coding genes [21]. More recently, TWBLV was detected in two Japanese pipistrelles in 2018 and 2020 [22]. In the same study, a fine-haired noctule bat (*Nyctalus plancyi velutinus*) was positive for lyssavirus, the first case of this species infected in Taiwan. This lyssavirus was genetically distinct from TWBLV and other lyssaviruses and was tentatively assigned TWBLV-2 [22]. In these studies, TWBLV was assigned to phylogroup I based on these preliminary genetic analyses; however, no attempts to characterize this virus antigenically in vivo or in vitro have been made thus far [21,22].

In an attempt to characterize this virus antigenically, a live recombinant virus, representative of the wildtype TWBLV, was generated. The lyssavirus glycoprotein (G) is the only viral protein on the virus surface and mediates virus-cell entry through its interaction with cell surface receptors. As a result, lyssavirus G is the sole target for neutralizing antibodies [23]. Previous studies utilizing reverse genetics techniques to exchange the RABV G of a vaccine vector with the G of another lyssavirus determined that these recombinant viruses were antigenically indistinguishable from the wildtype virus expressing the same G in the context of the humoral response [24,25]. In the present study, a recombinant virus containing TWBLV G was used to determine the ability of vaccine-derived sera and vaccine-induced antibodies to neutralize and confer protection against this virus.

## 2. Materials and Methods

### 2.1. Cells

BHK-21 (BHK; ATCC no. CCL-10) cells were propagated in Glasgow’s modified Eagle’s medium (GMEM; Gibco, Loughborough, UK) supplemented with 10% FBS, 100 U/mL penicillin, 100 µg/mL streptomycin and 25 U/mL mycostatin (Invitrogen, Paisley, UK). The BSR-T7/5 cells (BHK-derived cells that stably express T7 RNA polymerase—a gift from Dr Stefan Finke, FLI, Greifswald, Germany) were propagated in Dulbecco’s modified Eagle’s medium (DMEM; Gibco) supplemented with 5–10% FBS, 100 U/mL penicillin, 100 µg/mL streptomycin, and 25 U/mL mycostatin (Invitrogen).

### 2.2. Full-Length Plasmid Construction

Derived from the SAD B19 cDNA clone, the cDNA clone of the SN strain of RABV, cSN, was used as a DNA backbone vector as described previously [23,26,27]. Using Gibson Assembly techniques (New England Biolabs (NEB), Hitchin, UK) [28], the recombinant virus cSN-TWBLV was assembled by exchanging the cSN RABV G with the TWBLV G. TWBLV G open reading frame (ORF) (GenBank accession no. MF472710.1). The TWBLV G was codon optimized for expression in humans, synthesized, and subsequently cloned into the pI.18 expression vector (a gift from Carolyn Nicolson, NIBSC, UK) as it was initially used for pseudotype generation in human embryonic kidney cells. For insertion into cSN, the TWBLV G ORF was amplified with 20 bp overhangs directed at the 5′ and 3′ end of cSN using Q5^®^ Hot Start High-Fidelity 2X Master Mix (NEB) and the following primers: TWBLVfwd (5′-CTCAAAAGACCCCGGGAAAGATGCCCAATTACACACTG-3′) and TWBLVrev (5′-GTTGAAAGGCTAGCCAGTCCTCACGTATGGATTCGGGAC-3′). The underlined sequence within the primers represents the incorporated overhangs complementary to cSN. To generate a linear cSN excluding the RABV G, the following primers and the same PCR master mix were used: cSNFwd (5′- GGACTGGCTAGCCTTTCAAC-3′) and cSNrev (5′- CTTTCCCGGGGTCTTTTG-3′). Following the PCR, agarose gel electrophoresis was used to confirm the presence of amplicons of the appropriate size. The PCR purification was performed using the Monarch^®^ PCR & DNA Cleanup Kit (NEB), and residual vector DNA was restriction digested with DpnI (NEB). Using the NEBuilder^®^ Gibson Assembly Cloning Kit (NEB), the insert (TWBLV G) and vector (cSN) DNA was assembled. Following transformation, plasmid propagation and harvest, plasmids were sequenced before virus rescue was attempted.

### 2.3. Virus Rescue and Passage

Virus rescue was performed as described previously [25,29,30]. Briefly, 18 h before transfection, a 96-well plate (Corning) was seeded with 3 × 10^4^ BSR-T7/5 cells. Per well, 200 ng full-length genome plasmid and 40 ng of plasmids encoding the RABV N, P and L proteins were transfected using FuGENE^®^ 6 Transfection Reagent (Promega). Transfected cells were incubated for 72–96 h at 37 °C prior to further passage into a 12-well plate with fresh BSR-T7/5 cells. Further passage was undertaken, and cells were assessed for virus antigens, as described previously [31]. Final stocks of cSN-TWBLV were sequenced in their entirety using whole genome sequencing to confirm the expected sequence.

### 2.4. Virus Titration and Growth Kinetics

Titration of the virus was performed in BHK cells in triplicate, as described previously [26]. At 48 h post-infection (hpi), cells were fixed with 80% acetone and stained with FITC-conjugated anti-RABV N antibody (Fujirebio). Fluorescent foci were counted and recorded as fluorescent focus forming units per ml (ffu/mL).

The growth kinetics of both cSN and cSN-TWBLV were assessed using a multi-step growth curve, as described previously [24]. Cells were infected with the virus at a multiplicity of infection (moi) of 0.1. Following infection, 150 µL of media was removed per well at set time points (0 h, 6 h, 12 h, 24 h, 48 h, 72 h, 96 h, 120 h) and frozen at −80 °C. Each virus being assessed was independently grown in three wells, and each time point aliquot (three per time point) was titrated in triplicate. Titres were compared using the Mann–Whitney test.

### 2.5. In Vitro Studies

A modified version of the fluorescent-antibody virus neutralization test (mFAVN) was used to assess cross-neutralization, as described previously [32,33]. Excluding cSN-TWBLV, a panel of 9 polyclonal antisera and a panel of 13 viruses (Table 1) were used to assess cross-neutralization. This includes three RABV isolates: the RABV challenge standard virus-11 isolate (designated CVS in this study);,the RABV parent virus isolate used as the backbone for cSN-TWBLV (designated cSN in this study), and a wildtype or street RABV isolate (designated RABV in this study). In addition, three RABV-specific polyclonal sera, WHO serum, the WOAH serum, and hyperimmune human sera from a Human diploid cell vaccine (HDCV) (Rabies Vaccine BP; Pasteur Merieux, Lyon, France) recipient were used as standardised control sera. The titres for mFAVN were reported as reciprocal titres where a reciprocal titre of 1:16 typically neutralizes CVS equivalent to standard sera at 0.5 IU/mL since there are no internationally agreed cut-offs for other lyssaviruses. Three independently repeated mFAVN experiments were performed to give the mean titre values.

### 2.6. In Vivo Studies

All in vivo experimentation was carried out in ACDP3/SAPO4 biocontainment facilities at the Animal and Plant Health Agency (APHA), Weybridge, UK and complied with strict Home Office regulations under the Animals in Scientific Procedures Act (1986) and Home Office license PCA17EA73. Three-to-four-week-old BALB/c mice were purchased from a licensed and registered breeder within the UK (Charles River). On days 0 and 7 of the experiment, mice were vaccinated with x mL of the human rabies vaccine VeroRab (Novartis) via the intraperitoneal (ip) route. The mock vaccinated mouse group were vaccinated ip with MEM. The mice from the mock vaccinated and vaccinated group were challenged intracranially (ic) with 100 ffu/30 µL of cSN-TWBLV, 29 days postvaccination. Following the virus challenge, mice were observed twice daily for 22 days, and clinical signs were scored using a scale of 0–5 (where 0, no effect; 1, hunched body/ruffled fur; 2, limb twitching; 3 hindquarter paralysis; 4, progressive paralysis; 5, terminal recumbency/death) [42]. The pre-defined humane endpoint for termination was a clinical score of 1. At the end of the experiment, all mice were bled under terminal anaesthesia followed by cervical dislocation. Kaplan-Meier survival curves and the log-rank Mantel-Cox test were used to analyse survivorship rates of the single study.

### 2.7. Virus Detection

At the end of the study, a fluorescent antibody test (FAT) was undertaken, as described previously [43]. Histopathology and immunohistochemistry were performed, as described previously [44]. Mouse tissues were fixed in 10% neutral buffered formalin for two weeks before being sectioned coronally and processed into paraffin tissue blocks. Serial sections of 4 µm were prepared and stained with haematoxylin and eosin (H&E) for histopathology or immunohistochemistry for RABV N protein using a monoclonal antibody, mAb 5B12 (MyBioSource Inc., San Diego, CA, USA).

### 2.8. Molecular Analyses

At the end of the study, nucleic acids were extracted from brain tissue using Trizol (Invitrogen) according to the manufacturer’s instructions. To detect viral RNA in murine samples, a real-time SYBR-based reverse transcription PCR (RT-PCR) assay (Bio-Rad, Hemel Hempstead, UK) was used, as described previously [45,46]. The cDNA of CVS, at a known concentration (copies/uL), was diluted serially from 10^−1^ to 10^−8^ and used as a standard curve to interpolate the quantity of viral RNA in each sample tested by comparison across Ct values. Amplification using beta actin-specific primers was used as a control for RNA extraction as described previously [46]. Two mice from each group were tested in triplicate.

### 2.9. Serology

At 21 days post-vaccination, x mL of blood was collected from the dorsal vein of each mouse in CB300 blood collection tubes with a clotting activator (Sarstedt, Leicester, UK). Each serum sample was tested using a standard FAVN test to assess seroconversion to the VeroRab vaccine, as described previously [47]. Following the termination of the experiment, sera from mice that were cardiac bled under terminal anaesthesia was assessed by the FAVN against CVS and mFAVN against cSN-TWBLV. Mean virus-neutralizing antibody titres from three independently repeated tests were compared using two-way ANOVA and Tukey’s multiple comparisons tests.

### 2.10. Antigenic Cartography

Lyssavirus antigenic cartography maps were generated from the mFAVN neutralization data as described previously [30,32] using the following software: https://acmacs-web.antigenic-cartography.org/ (accessed on 29 January 2020) [48]. Whilst the map resolution increases with each increasing dimension, 3D maps were used for visualization and interpretation of antigenic distances as the increase in precision becomes negligible beyond three dimensions [32].

### 2.11. Analytical software

All graphs were generated and statistical analysis was performed using GraphPad Prism v8.4.2 (GraphPad Software, San Diego, CA, USA). For each data set, the appropriate statistical analysis function was selected.

## 3. Results

### 3.1. Virus Rescue and Titration

Using the RABV helper plasmids and cSN backbone, cSN-TWBLV was successfully rescued. To reach 100% infection of the cell monolayer, cSN-TWBLV required six passages, whereas cSN required two passages. Following the final passage of each virus, the virus was harvested and whole genome sequencing was used to compare the cSN-TWBLV virus sequence to the original cSN-TWBLV construct. The virus sequence showed 100% nucleotide identity to the construct (data not shown). Viruses were subsequently titrated where cSN exhibited a titre of 1.6 × 10^6^ and cSN-TWBLV exhibited a titre of 1.5 × 10^4^, however both were comparable to previous studies using virus constructs with a cSN vector [24,25,30].

### 3.2. Growth Kinetics

**Figure 1:**
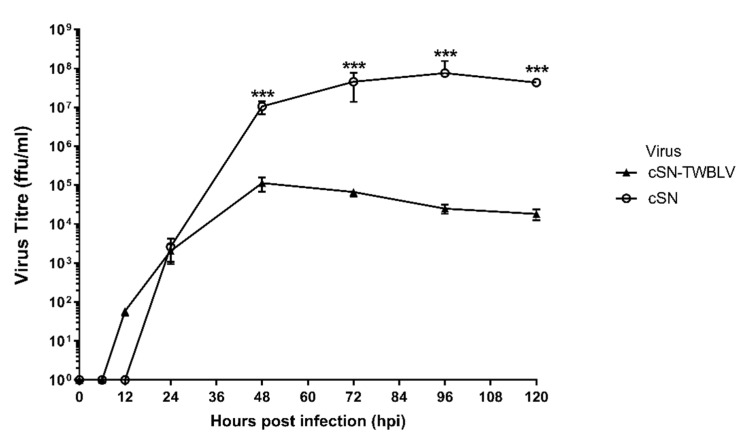
Growth kinetics of cSN-TWBLV and the vaccine backbone, cSN, in vitro. For each virus, BHK-21 cells were infected with an MOI of 0.1 to produce a multiple-step growth curve over the course of 120 h. The test was performed in triplicate and as three independent experiments where the mean and standard deviation of the results were plotted on a logarithmic scale. Asterisks indicate significant differences between the groups calculated using the Mann–Whitney test (*** *p* < 0.001).

Using multistep growth curves and due to inaccessibility to the TWBLV wildtype virus, the parent cSN virus was used to compare the growth kinetics of cSN-TWBLV (Figure 1). Both viruses were detected by 24 h at just over 10^3^ ffu/mL. Whilst cSN grew to a peak titre of 7.6 × 10^7^ ffu/mL at 96 hpi, cSN-TWBLV grew to a significantly lower peak titre of 1.13 × 10^5^ ffu/mL at 48 hpi (*p* < 0.001). The endpoint titre for cSN-TWBLV was 1.81 × 10^4^ ffu/mL over 3 log10 lower than cSN at 4.3 × 10^7^ ffu/mL (*p* < 0.001).

### 3.3. In Vitro Studies

In vitro studies to antigenically assess cSN-TWBLV were used to first determine the titre of RABV-specific standard sera required for neutralization above or equal to the pre-defined 0.5 IU/mL serological threshold. Following this, cross-neutralization between lyssaviruses was assessed.

#### 3.3.1. Assessment of cSN-TWBLV Neutralization Using Internationally Standardized Sera

**Figure 2:**
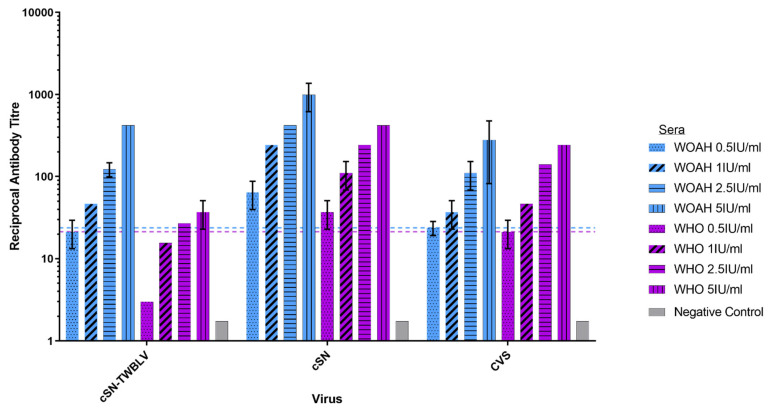
Neutralisation profiles of cSN-TWBLV, cSN and CVS against WOAH and WHO standard sera using a modified fluorescent antibody virus neutralisation (mFAVN) test. The test was performed in triplicate and as three independent experiments where the mean and standard deviation of the results were plotted on a logarithmic scale. The 0.5 IU/mL neutralisation cut-off is dictated by the serological standards against CVS (indicated by the coloured dashed lines–WOAH = blue, WHO = purple).

The neutralization profiles of cSN-TWBLV, cSN and CVS against increasing titres of standard sera, WOAH and WHO, were compared (Figure 2). Whilst all viruses were neutralised by serological standards, WHO sera showed the lowest titre of neutralizing antibodies against cSN-TWBLV, whilst WOAH sera demonstrated similar titres against cSN-TWBLV and CVS. For neutralization equivalent to 0.5 IU/mL of WHO and WOAH sera against CVS, 0.5 IU/mL of WOAH sera and 2.5 IU/mL of WHO sera were required to neutralise cSN-TWBLV. In addition, when comparing the two sera, the trend in levels of neutralizing antibodies was variable. WOAH and WHO sera showed a similar titre of neutralising antibodies against cSN and CVS. However, the two sera demonstrated differing neutralizing antibody titres against cSN-TWBLV, where WHO sera showed a reduction in neutralizing antibody titre compared to WOAH. Finally, both serological standards showed the highest titres against cSN.

#### 3.3.2. Ability of Phylogroup I-Specific Sera to Neutralize cSN-TWBLV

**Figure 3:**
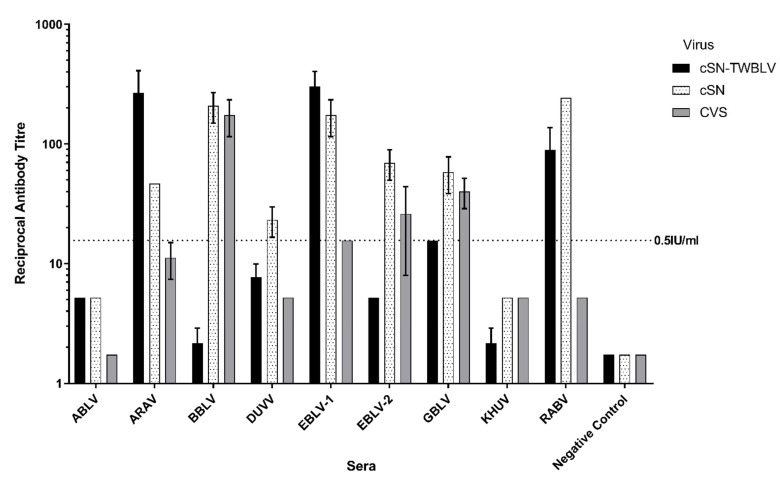
Cross-neutralization profiles of each phylogroup I lyssavirus-specific sera using an mFAVN test. The test was performed in triplicate and as three independent experiments where the mean and standard deviation of the results were plotted on a logarithmic scale. The 0.5 IU/mL neutralization cut-off is dictated by the WOAH sera against CVS (indicated by the dashed line). IRKV sera not shown.

To determine antigenic relationships between phylogroup I lyssaviruses and cSN-TWBLV, an mFAVN was used (Figure 3). Notably, the neutralization profiles of each virus tested were distinct, where cSN-TWBLV shows a very different profile to that of the parent vaccine strain cSN. Specific sera for ARAV and EBLV-1 exhibited the highest neutralising antibody titres against cSN-TWBLV, whilst specific sera for BBLV and wild/street RABV strain showed the highest neutralizing antibody titre against cSN. BBLV-specific sera also showed the highest neutralizing antibodies against CVS. Interestingly, BBLV-specific sera did not exhibit a neutralizing antibody titre above the 0.5 IU/mL cut-off against cSN-TWBLV. The virus cSN-TWBLV and CVS were least readily neutralized by the phylogroup I-specific sera panel as only four lyssavirus-specific sera exhibited neutralising antibodies above or equal to the 0.5 IU/mL cut-off. Conversely, cSN was the most readily neutralized as seven lyssavirus-specific sera exhibited neutralising antibodies above or equal to the 0.5 IU/mL cut-off. Whilst the wild/street RABV-specific serum neutralized cSN, it did not neutralize CVS, indicating possible antigenic divergence of wild/street strains to the cell culture-adapted CVS used regularly in diagnostic assays. Phylogroup II and III-specific sera exhibited no cross-neutralizing antibodies against CVS, cSN, and cSN-TWBLV (data not shown), and IRKV-specific sera were unavailable.

#### 3.3.3. Antigenic Cartography

**Figure 4:**
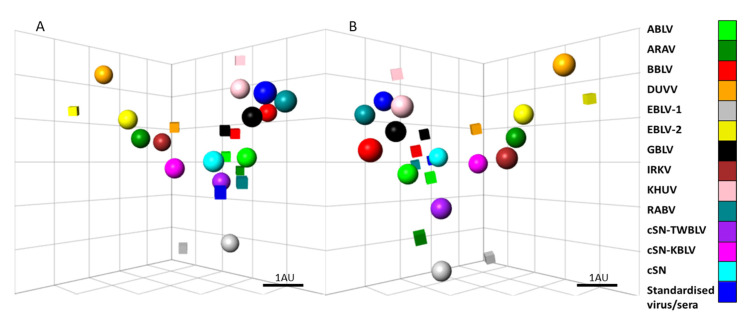
Three-dimensional antigenic map of phylogroup I lyssavirus and sera. (**A**) Viruses (spheres) and sera (boxes) are positioned such that the distance from each serum to each virus is determined by the neutralization titre. With the exception of the standardized virus (CVS) and standardized sera (WOAH), which are coloured dark blue, each phylogroup I virus and available associated specific sera have been colour coded according to the strain used. Multidimensional scaling is used to position both sera and viruses relative to each other, so the orientation of the map within the axes is free. Scale bar shows one antigenic unit (AU). (**B**) The same antigenic map, rotated to a different orientation for clarity.

The antigenic difference between isolates dictates the degree to which neutralizing antibodies induced by one isolate are effective against another. The cross-neutralization assay data were quantitatively assessed using antigenic cartography techniques described previously [32]. Results demonstrated that cSN-TWBLV was antigenically distinct from other phylogroup I lyssaviruses (Figure 4). Based on the antigenic distances on the 3D map, cSN-TWBLV was antigenically closest to ABLV (1.11 AU), cSN (1.58 AU), cSN-KBLV (1.59 AU), and EBLV-1 (1.63 AU).

### 3.4. In Vivo Vaccination Challenge Study

#### 3.4.1. Vaccination and Survival

**Figure 5:**
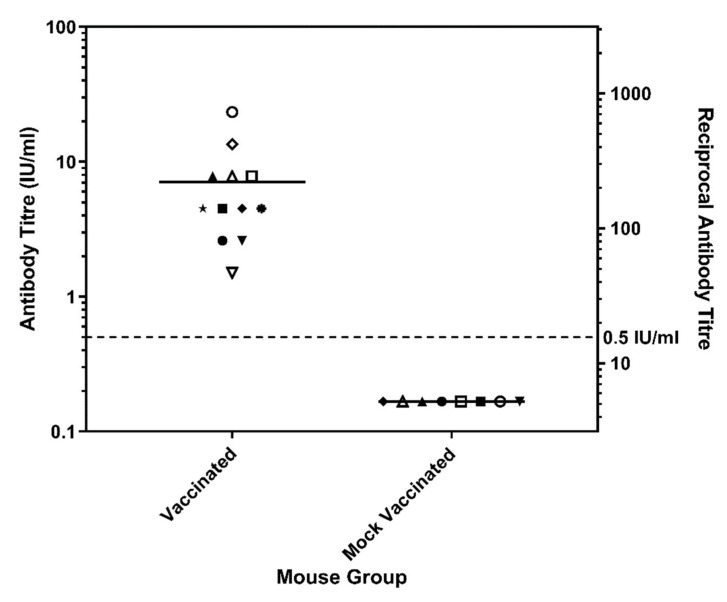
Post-vaccination serology on day 21 for mice vaccinated with VeroRab and mice mock vaccinated with MEM on days 0 and 7. All sera, each assigned a different symbol on the graph, were assessed for neutralizing antibodies by FAVN and plotted on a logarithmic scale.

To assess the ability of vaccine-derived immunity against cSN-TWBLV, mice were vaccinated with the rabies vaccine, VeroRab, and challenged with cSN-TWBLV as described. Twenty-one days after vaccination, a standard FAVN test was used to determine serological response. All vaccinated mice seroconverted to a titre above the internationally assigned cut-off for neutralization of RABV, 0.5 IU/mL, with serological responses ranging from 1.5 IU/mL and 23.4 IU/mL (Figure 5). On day 29 post-vaccination, mock vaccinated and vaccinated mice were challenged via the ic route with cSN-TWBLV. Zero mice from the vaccinated group (*n* = 12) and zero from the mock vaccinated group (*n* = 8) succumbed to infection, despite the mock vaccinated mice being seronegative post-mock vaccination. However, on days 10, 11, 13 and 15 post-challenge, mock vaccinated mice exhibited a slightly rough coated and hunched appearance but were not severe enough to be classified as a clinical score of 1 or 2, so they were not terminated. These mice recovered and showed no further clinical signs for the remainder of the experimental period.

#### 3.4.2. Serological Responses to Infection and Post-Vaccination Challenge

**Figure 6:**
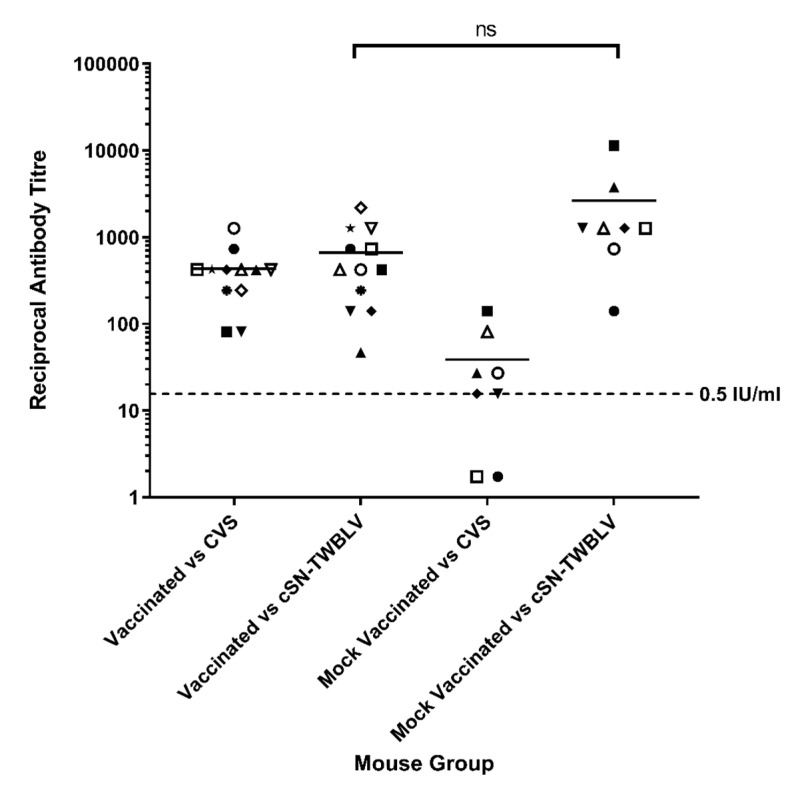
Serological evaluation of sera taken at termination cSN-TWBLV challenged mice were serologically assessed against CVS and cSN-TWBLV using FAVN and modified FAVN assays. The dotted line represents the 0.5 IU/mL antibody neutralization cut-off dictated by WOAH sera against CVS. Each symbol is representative of one animal. Scale is logarithmic. Significant differences between the groups were calculated using the Kruskal-Wallis test with Dunn’s multiple comparisons test (ns is not significant).

All animals were cardiac bled before being humanely terminated at the end of the experiment. Sera were assessed for seroconversion using both FAVN against CVS and mFAVN against the challenge virus. Mean titres were compared using the Kruskal-Wallis test with Dunn’s multiple comparisons test (Figure 6).

Sera from mock vaccinated mice (*n* = 8) were assessed: 75% (*n* = 6/8) had developed CVS-neutralizing antibody titres, and 100% (*n* = 8) developed significant cSN-TWBLV neutralising antibody titres, demonstrating productive infection and measurable humoral immune response to cSN-TWBLV. Additionally, the average neutralising antibody titres against cSN-TWBLV were significantly higher than the neutralising antibody titres against CVS at 1/2622 and 1/39, respectively (*p* < 0.001).

In the vaccinated group, the cSN-TWBLV challenge did not have a statistically significant effect on the serological titre when assessed for the ability to neutralize CVS versus cSN-TWBLV. However, the average neutralizing antibody titres against cSN-TWBLV were higher than CVS at 1/667 and 1/430, respectively.

Of the vaccinated and mock-vaccinated groups, the mock-vaccinated mice exhibited the highest average neutralizing antibody titres against cSN-TWBLV, but there was no significant difference from the vaccinated group (*p* = 0.734).

#### 3.4.3. Histopathology and Immunohistochemistry

Despite surviving to the end of the experiment, two mice from the mock vaccinated group were tested on the FAT and were negative for viral antigen (data not shown). Two mice from the same group were evaluated by histopathology and immunohistochemistry. Neither neuronal necrosis nor viral antigens were detected in the hippocampus or third ventricle (data not shown).

#### 3.4.4. Real-Time RT-PCR

RNA extracts from the brains of two mice from the two vaccine groups were evaluated by a SYBR real-time RT-PCR assay. The mice from the mock vaccinated groups had detectable RNA levels at Ct Values of 30.41 ± 0.57 and 32.57 ± 0.34, which equated to 7.64 × 10^2^ copies/µL and 1.88 × 10^2^ copies/µL, respectively. This indicates that whilst clinical disease did not develop, viral RNA was still present in the brain at the time of termination. The mice from the vaccinated group had no detectable viral RNA in the brain.

## 4. Discussion

Despite devastating health, social, and economic impacts, rabies remains a neglected tropical disease. Additionally, the continued encroachment of humans into more remote ecosystems has heightened the zoonotic threat of lyssaviruses from bats. The rabies vaccine and, in limited settings, rabies post-exposure immune globulins are the only tools against disease progression to a clinical incurable encephalitis following infection. However, protection against the more diverse members of the genus is limited. Consequently, the discovery of novel lyssaviruses warrants an in-depth investigation into genetic diversity, antigenicity, and vaccine efficacy.

Whilst TWBLV has been isolated and formally classified within the lyssavirus genus; only genetic classification has been previously investigated. The shared genome data of TWBLV enabled the synthesis of the TWBLV G ORF and subsequent generation of a cSN-TWBLV recombinant virus. In this study, the successfully rescued virus was able to replicate in vitro and further enabled antigenic characterization in the absence of wildtype TWBLV virus, through in vitro cross-neutralization assays and in vivo vaccination-challenge studies.

Growth kinetic assessment of this virus revealed that, despite successful virus rescue, endpoint titres were significantly different between cSN and cSN-TWBLV, with cSN-TWBLV reaching a lower titre (Figure 1). In addition, cSN-TWBLV peaked at 48 hpi with a titre of 1.13 × 10^5^ ffu/mL, whilst cSN peaked 48 h later with a titre of >10^7^ ffu/mL. This reduction in titre compared to the parent strain, cSN, may reflect sub-optimal protein interactions following interspecies G protein substitution. This could be due to impartial or incomplete interaction with heterologous M protein, leading to a sub-optimal assembly of G protein on the viral surface, although this hypothesis requires testing. Another hypothesis for decreased viral fitness is that the novel parent virus had even lower viral fitness within cell culture than the recombinant cSN-TWBLV virus. Whilst the wild-type virus has been isolated previously, viral fitness in cell culture has not been reported. In a previous study, wildtype EBLV-1 and -2 isolates grew to a lower titre than the recombinant cSN-EBLVs (referred to as SN-1 and SN-2 in the study), indicating that the low titres observed were not a result of the G protein-dependent processes such as receptor binding and viral entry, but rather a poor adaption for in vitro viral replication and assembly [24]. Finally, the G gene used was codon optimized for human expression. In previous studies, codon optimisation/de-optimisation of the RABV G gene decreased/slowed virus growth in vitro [49,50]. The effect of codon optimisation/de-optimisation on viral growth may greatly affect the growth of cSN-TWBLV in vitro and in vivo.

To assess the antigenicity of the TWBLV-G protein, mFAVNs were undertaken with a panel of sera specific for different lyssavirus G proteins. The amino acid sequence identities of TWBLV G protein and other lyssaviruses determined that EBLV-1 (AAX62856), EBLV-2 (AGG81481), KBLV (LR994545), ARAV (YP_007641395), and IRKV (YP_007641400) were the most closely related to TWBLV G with identities of 74.7%, 72.3%, 71.7%, 71.3%, and 70.6%, correlating with the data published by Hu et al., 2018. This also generally correlates to the cross-neutralization assays (Figure 3), as EBLV-1-specific sera showed the highest titre of neutralizing antibodies against cSN-TWBLV, and ARAV-specific sera showed the second highest titre of neutralizing antibodies. However, RABV-specific sera showed the third highest titre of neutralizing antibodies, and despite high sequence identity, EBLV-2-specific sera did not neutralize cSN-TWBLV above the 0.5 IU/mL cut-off. Finally, RABV-specific sera did not neutralize CVS above the 0.5 IU/mL threshold, indicating antigenic divergence between street RABV strains and CVS regularly used in diagnostic assays. This is concurrent with a previous study which determined laboratory-adapted RABV strains, such as CVS, to be antigenically distinct from a larger panel of WT-RABV strains [32]. In addition, the use of serological data to measure antigenic differences in cross-neutralization assays is limited by paradoxes or irregularities in the data, such as individual variations between sera raised against the same antigen or the difficulty of absolute quantification of sera raised against different isolates for different lyssavirus species [32].

Antigenic cartography was used, as described previously [30], to quantitatively analyse antigenic data. The cSN-TWBLV was positioned closest to ABLV, cSN, cSN-KBLV, EBLV-1, and IRKV with antigenic distances of 1.12 AU, 1.58 AU, 1.59 AU, 1.68 AU, and 1.85 AU, respectively (Figure 4). With the exceptions of ABLV and cSN, cSN-TWBLV positioned closest to the viruses that have the most similar amino acid percentage identity to TWBLV G. These distances, however, must be interpreted with caution as the resolution of these antigenic maps in the average prediction error have been previously determined to be 1.2 (SE, 0.17) antigenic units in 3D [32].

Due to the close antigenic and genetic relationship of EBLV-1 and IRKV to TWBLV, it was predicted that existing rabies vaccines would be able to confer protection against this virus. For EBLV-1, in vitro analyses have suggested that titres ≥4.5 IU/mL are required for neutralization [33]. In the present study, in vitro neutralization assays demonstrated that for neutralization of cSN-TWBLV, above the serological cut-off, an antibody titre of 2.5 IU/mL of WHO sera or 1.0 IU/mL of WOAH sera was required (Figure 2). WOAH standard sera showed a 1.1-fold higher neutralizing antibody titre against CVS compared to cSN-TWBLV. The different neutralization profiles of cSN, CVS, and cSN-TWBLV confirm the hypothesis that G is the dominant target for neutralizing antibodies. When using a standard challenge dose of 100 TCID_50_/50µL, these values only predict the conservative threshold for which in vivo protection against cSN-TWBLV is likely. As a result, an in vivo vaccination challenge study was performed.

Vaccinated mice survived to the end of the experiment; however, mock-vaccinated mice challenged with cSN-TWBLV also survived to the end of the experimental period (Figure 5). This is highly unusual as the cSN virus when inoculated ic causes clinical disease and death in this murine model [25], so this result suggested that the inclusion of a codon-optimized TWBLV G has attenuated the parental cSN virus.

Apathogenicity in vivo could be a result of the codon-optimisation of the TWBLV G. Previous studies have shown that codon-optimised/codon de-optimised lyssavirus G genes have reduced pathogenicity in vivo [49,50]. In this study, codon de-optimisation, paired with the observation that recombinant viruses traditionally show a decreased viral fitness to wildtype viruses, could form the hypothesis that the combination of these factors has resulted in reduced growth and decreased pathogenicity of cSN-TWBLV. However, without access to the live virus infectious viral isolate, this remains a supposition and needs to be determined in future studies.

Following the in vivo experiments in this study, the analyses of samples taken by necropsy revealed that a lyssavirus-specific antigen could not be detected in the brains of the cSN-TWBLV infected mice using FAT and IHC methods. However, viral RNA was detected in the cSN-TWBLV mock vaccinated mice by SYBR real-time RT-PCR, suggesting viral infection and subsequent clearance. In the cSN-TWBLV vaccinated mice, neither viral antigen nor viral RNA from the challenge virus was detected in the brains of mice. Previous studies have demonstrated that, post-infection, viral RNA levels are inversely proportional to virus-neutralizing antibodies [51,52]. Certainly, the mock vaccinated cSN-TWBLV infected mice that survived infection showed high levels of neutralising antibodies at the end of the experiment. Additionally, the cSN-TWBLV vaccinated mouse group had strong neutralizing antibodies post-vaccination but pre-challenge, which would have led to earlier viral clearance than the mock vaccinated control. These factors likely contribute to the outcomes observed through real-time RT-PCR testing of brain material as viral RNA in the cSN-TWBLV vaccinated group would have been cleared by 28 dpi. The serological assessment in this study also indicated that the cSN-TWBLV vaccinated group showed no increase in neutralizing antibody titres against the challenge virus versus the vaccine virus, whereas the cSN-TWBLV mock vaccinated group showed significantly higher neutralizing antibody titres against the challenge virus versus the vaccine virus, indicating lyssavirus infection and subsequent clearance in the mock vaccinated mice.

To conclude, the cSN-TWBLV construct was avirulent in vivo when inoculated by the ic route. Despite this, in vitro experiments indicated that vaccine protection with a minimum of 1 IU/mL is required for neutralization. Genetically, TWBLV G is closely related to EBLV- 1, EBLV-2, KBLV, ARAV, and IRKV. Cross-neutralization assays revealed that EBLV-1, ARAV, and RABV lyssavirus-specific sera showed the highest neutralizing antibodies against cSN-TWBLV and antigenic map data revealed that cSN-TWBLV clusters with ABLV, cSN, cSN-KBLV, EBLV-1, and IRKV. Further studies are required, preferably with a wild-type virus as a comparator, to examine the virulence of TWBLV.

## Figures and Tables

**Table 1:** Virus panel used and stock titre.

Designation	Lyssavirus Species	Polyclonal Antisera Used in this Study	Stock Titre (ffu/mL)	RV Number *	Isolated From	Year	Country	Genbank Accession Code ^$^	Reference
ABLV	Lyssavirus australis	Yes	1.5 × 10^5^	RV634	Bat	1996	Australia	AY062067 (G)	[34]
ARAV	Lyssavirus aravan	Yes	2.0 × 10^5^	RV3379	Bat	1991	Kyrgyzstan	EF614259	[35]
BBLV	Lyssavirus bokeloh	Yes	2.5 × 10^6^	RV2507	Bat	2009	Germany	JF311903	[36]
DUVV	Lyssavirus duvenhage	Yes	3.0 × 10^6^	RV131	Bat	1986	Zimbabwe	GU936870 (G)	[32]
EBLV-1	Lyssavirus hamburg	Yes	4.0 × 10^6^	RV20	Bat	1986	Denmark	KF155003	[37]
EBLV-2	Lyssavirus helsinki	Yes	4.3 × 10^4^	RV628	Bat	1996	UK	KY688136	[38]
GBLV	Lyssavirus gannoruwa	Yes	4.0 × 10^5^	RV3267	Bat	2015	Sri Lanka	KU244267	[39]
IRKV	Lyssavirus irkut	No	1.8 × 10^5^	RV3382	Bat	2002	Siberia	EF614260	[40]
KHUV	Lyssavirus khujand	Yes	5.0 × 10^4^	RV3380	Bat	2001	Tajikistan	EF614261	[35]
RABV	Lyssavirus rabies	Yes	1.6 × 10^5^	RV437	Raccoon Dog	-	Estonia	KF154997	[37]
CVS	Lyssavirus rabies	No	4.3 × 10^6^	Challenge Virus Standard-11 strain				EU352767	[41]
cSN	Lyssavirus rabies	No	1.2 × 10^6^	Recombinant virus; Street Alabama Dufferin(SADB19) backbone + SADB19 Glycoprotein				M31046.1 ^^^	[29]
cSN-KBLV	Not assigned	No	2.5 × 10^4^	Recombinant virus; Street Alabama Dufferin(SADB19) backbone + KBLV Glycoprotein				LR994545 ^£^	[5,30]

Abbreviations: ABLV, Australian bat lyssavirus; ARAV, Aravan virus; BBLV, Bokeloh bat lyssavirus; DUVV, Duvenhage virus; EBLV, European bat lyssavirus; GBLV, Gannoruwa bat lyssavirus; IRKV, Irkut virus; KHUV, Khujand virus; RABV, rabies virus. * Animal and Plant Health Agency lab identification number. ^$^ Where full genome sequence accession numbers cannot be found, glycoprotein sequence accession numbers have been included instead and are highlighted by (G). ^^^ SADB19 GenBank accession number. ^£^ KBLV GenBank accession number. Data not known.

## Data Availability

Not applicable.
